# Efficacy of Phacoemulsification combined with different auxiliary surgeries in the treatment of primary angle closure glaucoma and cataract: A retrospective study

**DOI:** 10.12669/pjms.42.4.14676

**Published:** 2026-04

**Authors:** Xiao Feng, Yanjing Liu, Yanbo Wang

**Affiliations:** 1 Xiao Feng Department of Ophthalmology, Zibo Center Hospital, Zibo, Shandong Province 255000, P.R. China; 2 Yanjing Liu Department of Ophthalmology, Zibo Center Hospital, Zibo, Shandong Province 255000, P.R. China; 3 Yanbo Wang Department of Endocrinology, Zibo Qidu Hospital, Zibo, Shandong Province 255000, P.R. China

**Keywords:** Cataract, Goniosynechialysis, Primary angle-closure glaucoma, Phacoemulsification, Trabeculectomy

## Abstract

**Objective:**

To evaluate and compare the clinical outcomes of phacoemulsification (PE) combined with trabeculectomy (TB) versus PE combined with goniosynechialysis (GSL) in managing primary angle-closure glaucoma (PACG) coexisting with cataract.

**Methodology:**

This clinical research was designed as a retrospective study, including 71 patients with PACG and cataract who underwent surgical treatment at Zibo Center Hospital between October 2023 and May 2025. Among them, 37 patients (37 eyes) underwent PE and intraocular lens implantation combined with TB (PE+TB group), and 34 patients (34 eyes) underwent PE and intraocular lens implantation combined with goniosynechialysis (PE+GSL group). Patients were followed up for 12 weeks after surgery to assess intraocular pressure (IOP), best-corrected visual acuity (BCVA), central anterior chamber depth (CACD), corneal endothelial cell counts (ECCs), and the incidence of postoperative complications.

**Results:**

After surgery, IOP, BCVA, CACD, and ECCs improved significantly from baseline in both groups. However, between-group comparison showed a statistically significant difference only in IOP, which was lower in the PE+TB group than in the PE+GSL group (P<0.05), whereas BCVA, CACD, and ECCs were comparable between groups (all P>0.05). During postoperative follow-up, the incidence of complications in the PE+GSL group was significantly lower than that in the PE+TB group (P<0.05).

**Conclusions:**

PE combined with either TB or GSL has its own advantages in the treatment of PACG with cataract: PE+TB results in lower IOP, while PE+GSL leads to fewer complications.

## INTRODUCTION

Primary angle-closure glaucoma (PACG) is one of the leading causes of irreversible blindness worldwide, especially in Asia. Its prevalence and blindness risk among elderly Asian populations are significantly higher than those of primary open-angle glaucoma.^1^ A study by Gupta et al.^1^ on Asian individuals aged 60-100 years showed that PACG is often diagnosed at an advanced stage with severe visual field damage. Another systematic review indicated that short axial length, shallow anterior chamber, increased lens thickness, as well as female gender and Asian ethnicity are important risk factors, suggesting that this disease has distinct high-risk population characteristics.^2^ The findings by Wang et al.^2^ emphasized the importance of early identification and intervention for high-risk anatomical structures and population features.

In recent years, numerous studies have confirmed that phacoemulsification (PE) combined with intraocular lens implantation can relieve angle crowding by deepening the anterior chamber and posteriorly displacing the iris root, resulting in sustained reduction of intraocular pressure (IOP). Atalay et al.^3^ reported that in eyes with primary angle closure and angle closure suspect, PE alone can significantly lower IOP and improve anterior segment structures even without concurrent glaucoma filtration surgery.^3^ In patients with concurrent PACG, further combination with goniosynechialysis (GSL) or other angle reconstruction surgeries is expected to restore the trabecular meshwork-Schlemm’s canal pathway while reducing long-term dependence on hypotensive medications. Real-world studies have demonstrated that PE combined with GSL generally achieves better surgical success rates and “surgical survival rates” during follow-up compared to some traditional filtration-related procedures, providing support for the “lens surgery + angle reconstruction” strategy.^4^,^5^

With the development of minimally invasive angle surgery, an increasing number of scholars have attempted to reconstruct the physiological aqueous outflow pathway through GSL, goniotomy, and other techniques, and have compared these approaches with traditional trabeculectomy. A study by Lin et al.^6^ found that in advanced PACG patients without cataracts, surgical peripheral iridectomy combined with GSL and goniotomy was non-inferior to trabeculectomy in terms of postoperative IOP control, while requiring fewer postoperative interventions. This suggests that the “angle reconstruction” strategy has the potential to become an alternative to classic filtration surgery.^6^ However, in angle-closure glaucoma patients with concurrent cataracts, direct comparative evidence regarding the relative advantages of PE combined with trabeculectomy versus PE combined with GSL (often accompanied by goniotomy) in terms of IOP-lowering efficacy, angle anatomical reconstruction, medication dependence, and complications remains lacking.^4^-^6^

In addition, glaucoma-related surgeries themselves can cause corneal endothelial cell loss, and the degree of endothelial damage varies among filtration surgeries, drainage device implantations, and combined cataract surgeries.^7^ Fang et al.^7^ noted that corneal endothelial cell density can decrease continuously after glaucoma surgery alone or combined with cataract surgery, which is particularly important for the PACG population with concurrent cataracts who may already have insufficient endothelial reserve. Therefore, when evaluating different combined surgical regimens, in addition to IOP and angle structure, corneal endothelial safety and postoperative complications are also important considerations influencing surgical selection. This retrospective study aims to assess the comparative efficacy of PE combined with TB or GSL in the treatment of patients with PACG with cataract.

## METHODOLOGY

This clinical research was designed as a retrospective study. A computerized retrieval system was utilized to screen the Electronic Medical Records (EMR) of all patients with PACG and cataract who received surgical intervention at Zibo Center Hospital between October 2023 and May 2025. By strictly adhering to the pre-defined inclusion and exclusion criteria, all eligible consecutive patients were included in the final analysis without any subjective or selective exclusion. A total of 71 patients (71 eyes) met the criteria, comprising 37 eyes in the PE+TB group and 34 eyes in the PE+GSL group.

### Ethical Approval:

The ethics committee of Zibo Center Hospital approved this study (Approval No. 2025-277; Date: October 31, 2025). The requirement for informed consent was waived by the Ethics Committee due to the retrospective nature of the study and the use of strictly anonymized clinical data.

### Inclusion criteria:

- Meets the diagnostic criteria for PACG with cataract.^1^
- Age ≥ 18 years old.
- The above-mentioned joint surgery has been completed, and the clinical data are complete.

### Exclusion criteria:

- Combined ocular trauma.
- Accompanied by other eye diseases.
- Previously had secondary angle closure glaucoma.
- Patients who have undergone the above-mentioned combined surgery in the past, as well as other patients who have undergone eye surgery.

### Surgical methods:

Preoperatively, all patients received antibiotic eye drops for infection prophylaxis, along with topical IOP-lowering therapy and dehydration therapy. All surgeries were performed by a single senior surgeon following a strictly standardized surgical protocol to ensure high internal consistency and comparability of intraoperative steps.

### PE Combined with Intraocular Lens implantation:

Standard phacoemulsification with intraocular lens implantation was performed in all eyes. After a clear corneal incision was made, ophthalmic viscoelastic material was injected into the anterior chamber, followed by routine phacoemulsification and implantation of an intraocular lens in the capsular bag.

### For patients undergoing PE combined TB:

A 1×1 mm tissue block was excised from the posterior edge of the scleral tunnel using a Kelly Descemet’s membrane punch. Peripheral iridectomy was performed with Vannas scissors. The scleral flap was secured with two 10-0 nylon sutures, and the conjunctiva was meticulously closed in a watertight fashion at the limbus using 7-0 Vicryl sutures. Physiological saline was injected into the anterior chamber through a side port to observe the formation of diffuse conjunctival blebbing and any potential leakage.

### For patients undergoing PE combined GSL:

This procedure consistently included excisional goniotomy with the KDB following goniosynechialysis in all cases to ensure surgical uniformity. Initially, gentle radial pressure was applied toward the pupillary center to separate peripheral adhesions and expose the trabecular meshwork. Subsequently, under direct gonioscopic guidance, a standardized ‘push-pull’ technique was employed to cleanly and completely resect the trabecular meshwork over a uniform range of 120–150 degrees in the nasal angle. This standardized approach ensured consistent exposure of the Schlemm’s canal across the entire cohort. Postoperative care included standard antibacterial prophylaxis for one week, and anti-inflammatory therapy was gradually tapered over four weeks.

### Data collection time points:

Data were collected preoperatively as well as at 12 weeks postoperatively. The central anterior chamber depth (CACD), defined as the distance from the corneal epithelium to the anterior lens surface, was measured using optical coherence biometry (IOL Master, Carl Zeiss Meditec) by an experienced technician. Other parameters included age, gender, eye laterality, IOP, BCVA, corneal endothelial cell counts (ECCs), and complications.

### Statistical methods:

SPSS/PC statistical software (version 26.0; IBM Corp, Armonk, NY, USA) for Windows was used for all analyses. The count data were presented as n (%), and the difference between groups was calculated using Fisher’s exact test. The measurement data were presented as mean ± standard deviation (SD). Independent sample t-test was used to compare the differences between the two groups, and paired t-test was used for intra group comparison before and after. P<0.05 indicated statistically significant difference.

## RESULTS

In this retrospective study, clinical records of 71 patients (71 eyes) were included. There were 29 males and 42 females in the cohort, with the ages ranging from 55 to 82 years (an average of 70.5±6.4 years). A total of 37 patients (37 eyes) underwent PE combined with TB (PE+TB group), and 34 patients (34 eyes) underwent PE combined with GSL (PE+GSL group). There was no significant difference in baseline data between the two groups (P>0.05) (Table-I).

**Table-I:** Comparison of baseline data between two groups.

Baseline data	PE+TB (n=37)	PE+GSL group (n=34)	χ^2^/t	P
Sex, n(%)			0.832	0.470
Male	17 (45.9)	12 (35.3)		
Female	20 (54.1)	22 (64.7)		
Age (years)	71.7±6.1	69.1±6.6	1.734	0.087
Eye, n(%)			1.024	0.436
Left	28 (75.7)	22 (64.7)		
Right	9 (24.3)	12 (35.3)		
Baseline IOP (mmHg)	28.3±11.4	26.7±11.4	0.576	0.567
Baseline BCVA (logMAR)	0.68±0.15	0.71±0.15	-0.929	0.356

***Note:*** PE: phacoemulsification; TB: trabeculectomy; GSL: goniosynechialysis; IOP: intraocular pressure; BCVA: best corrected visual acuity.

There was no statistically significant difference in baseline IOP, BCVA, CACD, and ECCs between the two groups (P>0.05). After surgery, IOP, BCVA, CACD, and ECCs improved significantly compared with baseline in both groups. Between-group comparison demonstrated that only postoperative IOP differed significantly, being lower in the PE+TB group than in the PE+GSL group (P<0.05), while BCVA, CACD, and ECCs showed no significant difference between groups (all P>0.05) (Table-II). In terms of complications, the incidence rate of the PE+GSL group (2.94%) was significantly lower than that of the PE+TB group (18.92%) (P<0.05) (Table-III).

**Table-II:** Comparison of IOP, BCVA, CACD, and ECCs count between two groups.

Variables	PE+TB (n=37)	PE+GSL group (n=34)	t	P
***Baseline***				
IOP (mmHg)	28.3±11.4	26.7±11.4	0.576	0.567
BCVA (logMAR))	0.68±0.15	0.71±0.15	-0.929	0.356
CACD (mm)	2.17±0.23	2.10±0.22	1.508	0.136
ECCs (cells/mm^2^)	2557±320	2639±328	-1.061	0.292
***After surgery***				
IOP (mmHg)	10.7±2.9*	12.4±2.3*	-2.618	0.011
BCVA (logMAR))	0.28±0.11*	0.27±0.09*	0.565	0.574
CACD (mm)	4.07±0.31*	4.04±0.26*	0.476	0.635
ECCs (cells/mm^2^)	2072±364*	2021±300*	0.649	0.519

***Note:*** Compared with the baseline of the same group,

* P<0.05. PE, phacoemulsification; TB, trabeculectomy; GSL, goniosynechialysis; IOP, intraocular pressure; BCVA, best corrected visual acuity; CACD, central anterior chamber depth; ECCs, endothelial cell counts.

**Table-III:** Comparison of incidence of complications between two groups.

Complication	PE+TB (n=37)	PE+GSL group (n=34)	P
Corneal edema	1 (2.70)	0 (0.00)	-
Shallow anterior chamber	2 (5.41)	0 (0.00)	-
Fibrinous iris exudation	1 (2.70)	0 (0.00)	-
Macular edema	1 (2.70)	0 (0.00)	-
Hyphema	2 (5.41)	1 (2.94)	-
Overall incidence rate	7 (18.92)	1 (2.94)	0.024

In terms of complications, the overall incidence in the PE+GSL group (2.94%, 1/34) was significantly lower than that in the PE+TB group (18.92%, 7/37) (P=0.024). These complications were categorized based on their severity and the necessity for clinical intervention. Transient complications included mild corneal edema (one in PE+TB) and minor hyphema (Two in PE+TB, one in PE+GSL), all of which resolved completely within one week postoperatively through standard topical anti-inflammatory therapy and observation. In the PE+TB group, one instance of fibrinous iris exudation completely resolved within five days post-treatment with intensified topical corticosteroids. Clinically significant complications, observed only in the PE+TB group, included two cases of Grade-I shallow anterior chamber and one case of macular edema. The shallow anterior chambers returned to normal depth within one week after treatment with topical mydriatics and pressure patching, without requiring anterior chamber reformation or involving malignant glaucoma. The case of macular edema remained stable at the 12-week follow-up without additional surgical intervention. Notably, no severe complications requiring surgical re-intervention in the operating room were observed in either group.

## DISCUSSION

This study directly compared the short-term outcomes of PE combined with TB versus PE combined with GSL in patients with PACG and cataract. Our primary findings demonstrate that both surgical approaches significantly IOP, visual function, and anterior segment parameters from baseline. PE+TB achieved a significantly lower terminal IOP at 12 weeks, whereas PE+GSL was associated with a markedly lower incidence of postoperative complications.

These results align with established evidence from regional and international studies.^8^-^11^ Regionally, our findings regarding significant visual improvement and IOP reduction are consistent with reports from Pakistani cohorts; for instance, Bodla et al.^9^ observed that cataract extraction significantly improves visual outcomes and refractive indices. Internationally, this pattern mirrors the overall findings of studies by White et al.^8^ and Gupta et al.^10^, which suggest that lens surgery combined with internal angle reconstruction (such as GSL and goniotomy) provides stable IOP reduction and controllable risks.^8^,^10^,^11^ However, when a lower target IOP is required, the filtration pathway (TB) offers a higher “ceiling” for pressure reduction despite its higher risk of complications and management costs.^8^-^11^ The anatomical rationale for prioritizing lens extraction stems from its capacity to directly mitigate anterior segment crowding; this approach provides more predictable expansion of the angle geometry compared to standalone filtration procedures.^12^

The discrepancies in therapeutic efficacy between studies can be attributed to several mechanistic factors. Evidence based on quantitative parameters suggests that while standalone PE achieves initial angle opening, additional GSL or goniotomy further improves aqueous outflow facility in specific patients.^13^,^14^ However, this add-on effect is heterogeneous. Comparative studies have shown that baseline peripheral anterior synechiae (PAS) extent, disease duration, and the reversibility of trabecular function significantly stratify benefits.^8^,^10^,^13^,^14^ In cases of extensive and long-standing PAS, the response of functional outflow channels remains limited even after anatomical lysis, explaining why the difference between PE+GSL and standalone PE may not be stable in all cohorts.^8^,^13^,^15^ Conversely, in patients with moderate PAS burden and reconstructible angles, the probability of achieving greater IOP reduction is higher, as reported in several multicenter series.^8^,^9^,^16^

Regarding safety, the distinct profiles of these approaches are consistent with previous literature.^15^,^16^ The most common complications of internal angle reconstruction are transient hyphema and corneal edema, which typically resolve with conservative treatment.^15^,^16^ In contrast, the risks associated with the filtration pathway in angle-closure eyes are more dependent on anterior segment dynamics. Ultrasound biomicroscopy (UBM) studies have revealed that postoperative ciliary body anterior displacement is a key driver of complications like malignant glaucoma, consistent with the higher complication burden in our PE+TB group.^17^,^18^ Furthermore, both groups showed comparable, non-clinically significant decreases in ECCs, aligning with evidence that endothelial changes are limited in the short term for combined cataract-angle surgery.^17^,^18^

Compared with relevant studies, this study’s strengths lie in its direct comparison that aligns with clinical decision-making and its multidimensional assessment. The comparison groups represent the two main clinical approaches used in practice, providing strong external validity.^8^,^11^ By simultaneously evaluating anatomical (CACD), functional (IOP), and safety (ECCs) parameters, this research provides a complete “anatomical remodeling → functional improvement → risk cost” evaluation chain.^9^,^12^,^18^ Furthermore, the standardization of procedures within our cohort reduces confounding factors from technical variations.^16^-^20^ Nevertheless, future research should prioritize long-term follow-up beyond 12 weeks to confirm long-term stability and late-onset complications. Incorporating standardized quantitative angle imaging (AS-OCT/UBM) as a routine tool is essential for providing precise mechanistic evidence. Finally, large-scale multicenter randomized controlled trials are needed to validate these results through stratified analyses based on PAS burden.

### Limitations:

First, the retrospective design, small sample size (N=71), and short 12 weeks follow-up limit the evaluation of long-term IOP stability, bleb function, and late-onset complications. Consequently, these early findings cannot be fully extrapolated to long-term outcomes. Second, although baseline characteristics were comparable (all P > 0.05), the analysis relied on univariate tests; multivariate modeling was avoided to prevent overfitting due to sample size constraints. Furthermore, inter-center variations in learning curves and perioperative medication strategies may affect generalizability. Third, the lack of standardized preoperative quantification of PAS extent—such as exact degrees of adhesion—hinders the interpretation of GSL efficacy, particularly in patients with irreversible trabecular damage. Finally, the individualized nature of post-operative medication adjustments precluded a rigorous quantitative comparison of anti-glaucoma drug reduction.

## CONCLUSION

For patients with PACG complicated with cataracts, both PE+TB and PE+GSL can significantly reduce IOP and improve visual function and anterior segment anatomy in the short term. However, PE+TB achieves lower terminal IOP at 12 weeks, whereas PE+GSL is associated with significantly fewer complications. Due to the limited follow-up duration, these findings represent only early surgical outcomes and should not be interpreted as definitive long-term results. Further prospective studies with extended follow-up of one to eight years and larger sample sizes are essential to verify the long-term benefits and population applicability of both approaches.

### Recommendations:

Future multicenter, prospective randomized trials with extended follow-up and standardized imaging such as UBM are essential to validate these findings.
